# Redefining creativity in AI era: Bloom’s taxonomy framework and peer interaction elevate higher-order thinking skills

**DOI:** 10.1371/journal.pone.0356609

**Published:** 2026-10-14

**Authors:** Abdhy Aulia Adnans, Noor Raihan Ab Hamid, Norlina Kamudin, Andri Dayarana K. Silalahi

**Affiliations:** 1 Vocational School, Universitas Padjadjaran, Sumedang, Indonesia; 2 Yayasan Bhakti Pertiwi Jaya, Jakarta, Indonesia; 3 Department of Marketing and Logistics Management, College of Management, Chaoyang University of Technology, Taichung, Taiwan; Hunan Normal University, CHINA

## Abstract

Artificial intelligence is transforming cognitive learning by reshaping pedagogical models and raising questions about its impact on higher-order thinking. AI-driven personalized learning improves knowledge acquisition, but its effect on creativity remains largely unexplored. Most research emphasizes content delivery over cognitive engagement, which leaves gaps in understanding how AI influences deeper learning processes. This study investigates how ChatGPT-enabled personalized learning, framed through Bloom’s Taxonomy, influences creative thinking among Indonesian higher education students, considering the moderating role of peer interaction. Using SEM via Smart-PLS 4.1, data from 533 Indonesian students reveal that personalized learning strengthens both lower-order cognitive skills—such as remembering, understanding, and applying—and higher-order functions, including analyzing, evaluating, and creating, which are essential for creativity. Among these, analyzing and creating have the strongest impact on creative thinking. Peer interaction selectively moderates this relationship, significantly enhancing creativity in tasks involving creation, while its effect on other cognitive functions remains limited. This study introduces a dynamic framework that integrates personalized learning, Bloom’s Taxonomy, and peer collaboration, offering new insights into how tailored cognitive processes and selective peer engagement drive creativity in learning environments.

## 1. Introduction

Artificial intelligence is no longer a supplementary tool in education but a transformative force reshaping cognitive learning. It challenges traditional pedagogical approaches and raises critical questions about its role in fostering higher-order thinking skills [1,2]. While widely adopted for personalized learning, its influence on developing skills like creative thinking and problem-solving remains underexplored [3,4]. Prior research has primarily focused on AI’s function in knowledge dissemination rather than its potential to cultivate critical and creative cognition [5,6]. As the AI-driven low-cost demands greater cognitive adaptability, it is essential to investigate AI’s role in higher-order thinking beyond knowledge acquisition.

Bloom’s Taxonomy outlines six stages of learning, from basic knowledge recall to advanced skills like evaluation and creation [7]. Understanding AI’s potential in cognitive development requires examining how these structured learning stages align with AI-driven educational tools. AI tools such as ChatGPT have shown potential in guiding students through these stages by offering instant feedback, encouraging discussion, and improving problem-solving abilities [8,9]. While AI’s impact on cognitive development is widely acknowledged, its role in collaborative learning remains unclear. Working with peers enhances engagement and helps build knowledge, but little research has explored how AI-driven personalization can support these interactions [10,11]. Further study is needed to understand how AI, structured learning models, and peer collaboration together influence creative thinking [12,13].

Despite the integration of AI into education, there remains a significant gap in understanding how AI facilitates higher-order cognitive skills, particularly creative thinking [1,4]. Current research primarily focuses on AI’s contributions to knowledge acquisition, neglecting its potential to enhance critical engagement and creativity [2,9]. The role of AI in fostering peer collaboration and dynamic knowledge co-construction is similarly underexamined [6,10]. While AI systems provide interactive learning experiences, their ability to support creativity in structured cognitive frameworks such as Bloom’s Taxonomy remains unverified [5,6]. Moreover, creative thinking is an essential skill in an AI-driven society, yet research lacks insight into how AI-driven platforms can cultivate this capability effectively [14,15]. Addressing this research gaps is imperative for leveraging AI’s full potential in education.

While prior research highlights the benefits of ChatGPT in educational settings, there is still a substantial gap in understanding its role in promoting higher-order cognitive skills, particularly creative thinking. Mogavi et al. [1] underscored ChatGPT’s contribution to improving engagement and self-efficacy through personalized learning experiences. However, their study primarily emphasized foundational cognitive skills such as knowledge retention and comprehension rather than how ChatGPT facilitates cognitive progression to synthesis and evaluation. This limitation leaves open critical questions regarding AI’s capacity to cultivate deep learning processes. Additionally, concerns about over-reliance on AI tools persist, with scholars warning that without proper scaffolding, students may passively consume AI-generated knowledge rather than actively construct their own understanding [2,3]. The absence of studies addressing how AI fosters cognitive complexity beyond rote learning presents a pressing need for further research on its role in higher-order thinking development within Bloom’s Taxonomy.

The role of ChatGPT in fostering collaborative learning and dynamic problem-solving remains another underexplored dimension. While DeMara et al. [9] introduced the STEM-Optimal Digitized Assessment Strategy (SODAS) to enhance cognitive outcomes through structured assessments aligned with Bloom’s Taxonomy, the framework lacked the real-time adaptability that AI systems like ChatGPT can offer. Unlike static feedback mechanisms, ChatGPT’s ability to provide instant, personalized responses fosters deeper engagement in scenario-based learning and interactive knowledge construction [5,6]. However, existing studies fail to account for how ChatGPT mediates peer collaboration and collective cognitive amplification in educational settings [10,11]. Similarly, research by Lijie et al. [13] examined AI’s role in creativity but was limited to vocational learners, overlooking the interactive and dialogical aspects of AI-enhanced peer collaboration. Addressing this gap requires examining how ChatGPT facilitates structured dialogue, knowledge co-creation, and creative problem-solving in collaborative learning environments. This study seeks to bridge these gaps by integrating ChatGPT-driven adaptive learning with peer collaboration to enhance creative thinking across Bloom’s hierarchical levels.

This study is motivated by the increasing demand for educational frameworks that move beyond knowledge transmission to cultivate higher-order cognitive skills, particularly creativity, critical thinking, and problem-solving [8,16]**.** With AI’s growing role in personalized learning, it is crucial to explore how tools like ChatGPT can dynamically support students in navigating Bloom’s cognitive hierarchy while fostering interactive peer engagement [10,17]. Despite the widespread implementation of AI-driven educational interventions, there remains limited empirical research on how these technologies can facilitate structured, creative learning pathways that promote both individual cognitive development and collaborative problem-solving [6,14]. Addressing this gap is essential for maximizing AI’s potential in educational settings.

This study aims to establish a comprehensive model that integrates ChatGPT-driven personalized learning with Bloom’s Taxonomy and peer collaboration to enhance creative thinking. By leveraging AI’s real-time adaptability and interactive learning capabilities, the research examines how ChatGPT fosters structured cognitive progression, guiding learners from foundational knowledge acquisition to creative synthesis [9,15]. Additionally, the study explores the moderating effect of peer collaboration in amplifying cognitive engagement and creative problem-solving [12,13]. The findings are expected to provide critical insights for educators and policymakers in designing AI-enhanced learning environments that optimize creativity and higher-order thinking skills, addressing pressing challenges in contemporary education [2,3].

The growing use of ChatGPT in personalized learning raises key questions about its impact on cognitive development beyond memorization. While studies show its effectiveness in reinforcing knowledge retention and comprehension [1,2], these benefits are mostly seen in lower-order thinking. Bloom’s Taxonomy emphasizes that true mastery requires advancing to analysis, synthesis, and creative problem-solving [4,14]. AI can personalize learning, but it remains unclear whether this fosters deeper cognitive growth or merely optimizes knowledge recall. Without empirical validation, there is a risk that AI reinforces passive learning rather than promoting creative thinking [3]. This raises a crucial question: *Can ChatGPT-driven personalized learning support higher-order cognitive skills within Bloom’s Taxonomy, particularly creativity?*

Beyond individual learning, peer collaboration has long been recognized as a driver of deep cognitive engagement, allowing learners to refine their understanding through discussion, debate, and collective problem-solving [10,11]. However, the integration of AI as a mediating agent in collaborative learning environments remains underexplored. Traditional models of peer learning assume human-to-human interaction as the primary vehicle for knowledge construction, overlooking how AI-generated feedback can dynamically influence discourse [12]. ChatGPT’s ability to introduce diverse perspectives, challenge assumptions, and provide iterative, context-sensitive responses introduces a new layer of interactivity that may transform traditional peer learning structures. Yet, without clear empirical evidence, it is uncertain whether AI-driven interventions enhance collaboration or create dependency on machine-generated insights, potentially reducing students’ agency in cognitive co-construction [9,13]. This ambiguity necessitates further exploration into AI’s role in shaping collective intellectual growth. Therefore, this study proposes: *In what ways does peer collaboration moderate the relationship between ChatGPT-driven personalized learning and the development of higher-order thinking skills?*

This study contributes to the advancement of AI-driven pedagogy by critically assessing whether ChatGPT enhances or impedes deep learning through personalization and peer interaction. Theoretically, this study expands existing frameworks on Bloom’s Taxonomy by incorporating AI-driven adaptive learning into the discourse on higher-order cognitive development. Practically, the findings offer insights for educators seeking to design AI-enhanced learning environments that balance personalization with interactive, student-driven learning, addressing the pressing need for AI systems that support—not replace—human cognitive engagement.

## 2. Literature review

### 2.1. Previous studies and gaps

The integration of ChatGPT into personalized learning environments has demonstrated significant potential to enhance educational outcomes particularly its significance in achieving Bloom’s Taxonomy as shown in Table 1. Mogavi et al. [1] highlighted its early adoption across various educational contexts, stressing improved engagement and self-efficacy through content personalized to individual needs. While their study demonstrated benefits in knowledge acquisition and comprehension, it did not fully address ChatGPT’s role in fostering higher-order thinking skills such as synthesis and evaluation. Additionally, concerns about over-reliance on AI tools were raised, with risks of students becoming passive learners rather than active participants in knowledge construction [2,3]. This gap highlights the need for further research into how AI-driven personalization supports cognitive progression and active learning to achieve higher order level thinking from Bloom Taxonomy.

**Table 1:** Previous studies and gaps.

Reference	Context	Objective	Theories / Concepts	Contribution
Mogavi et al. [1]	Early adopters’ use of ChatGPT in education.	To analyze ChatGPT’s benefits and challenges in education.	Human-Computer Interaction, AI in Education	Provides insights into applications and guides future research.
DeMara et al. [9]	STEM-Optimal Digitized Assessment Strategy (SODAS).	To enhance learning outcomes using scalable personalized feedback.	Bloom’s Taxonomy, Metacognition	Highlights strategies for improving cognitive skills with technology.
Akpur [4]	Critical, reflective, and creative thinking in digital learning.	To examine their interaction and impact on academic achievement.	Critical, Reflective, and Creative Thinking	Demonstrates higher-order thinking’s role in promoting academic success.
Lijie et al. [13]	Vocational learners using ChatGPT.	To explore how critical thinking mediates motivation and creativity.	Cognitive Flexibility Theory	Shows critical thinking’s role in linking motivation and creativity with AI tools.
Yao et al. [12]	Sustainable teaching innovation in graduate education.	To study teaching innovation’s effects mediated by self-efficacy.	Organizational Creativity Theory	Emphasizes teaching innovation’s role in fostering creativity.
This Study	ChatGPT-driven personalized learning in higher education.	To examine ChatGPT’s impact on cognition and creativity.	Bloom’s Taxonomy, Collaborative Learning	Proposes a new model integrating personalized learning, peer collaboration, and cognitive progression.

Building on the potential of personalized feedback, DeMara et al. [9] introduced the STEM-Optimal Digitized Assessment Strategy to improve cognitive and metacognitive outcomes by aligning assessments with Bloom’s Taxonomy. However, the framework relied on static and predefined assessments and did not incorporate the dynamic adaptability that AI tools like ChatGPT can provide. Unlike static systems, ChatGPT provides real-time feedback and personalized guidance that deepen engagement with tasks like scenario-based learning and problem-solving [5,6]. While SODAS showed scalability in assessment strategies, it lacked focus on collaborative learning contexts where ChatGPT could mediate peer discussions and foster creative problem-solving [10,11].

Similarly, Akpur [4] explored the relationships among critical, reflective, and creative thinking skills, emphasizing their importance for academic success. However, the study did not consider how AI-driven personalization could enhance these cognitive skills by adapting to learners’ needs and supporting real-time critical engagement. For example, ChatGPT can present diverse perspectives and prompt evaluative reasoning, which may strengthen students’ analytical and reflective abilities as they progress through Bloom’s hierarchy [14,15]. This gap calls for research on how tools like ChatGPT can facilitate both individual cognitive growth and collaborative knowledge building.

More recently, Lijie et al. [13] explored how ChatGPT supports creativity by mediating the relationship between motivation and critical thinking. While their findings demonstrate AI’s role in fostering motivation-driven creativity, the study narrowly focused on individual learners in vocational settings, overlooking the collaborative and interactive dimensions of learning that are essential in higher education. Similarly, Yao et al. [12] investigated the effects of sustainable teaching innovations on creative thinking, mediated by playfulness and academic self-efficacy. Although their study highlights the value of innovative pedagogy, it underutilized the potential of AI tools to integrate personalized feedback and collaborative dynamics into teaching frameworks. Thus, the importance of how AI-driven personalized learning were underutilized yet it can bridge individual and collaborative cognitive processes.

In addition to student centered evidence, recent educator centered research indicates that generative AI integration in higher education is shaped by institutional governance and psychosocial conditions. Qualitative studies of Palestinian university educators describe tensions between opportunity and precarity, including policy uncertainty, academic integrity concerns, and the need to preserve learner agency when generative AI is used for teaching and learning tasks [18,19]. Complementary adoption research grounded in an extended unified theory of acceptance and use of technology identifies factors that shape educators’ acceptance of generative AI, emphasizing perceived usefulness and enabling conditions [20]. Educators also report technostress and emerging dependency risks, suggesting that sustainable use requires support structures and boundaries that protect professional judgement and student agency [21,22]. These findings provide contextual grounding for the present student centered model by clarifying why instructional design and meaningful human interaction remain central when AI driven personalization is introduced into higher order and creativity oriented learning.

This study addresses key gaps by introducing a framework that combines ChatGPT-driven personalized learning with Bloom’s Taxonomy to enhance creative thinking skills. Unlike earlier studies that focused on isolated cognitive dimensions or individual outcomes, this research takes a broader approach by integrating collaborative cognitive amplification as a moderating factor. ChatGPT’s adaptive features, such as personalized feedback and diverse perspectives, help learners move systematically through Bloom’s hierarchy, from basic understanding to creative synthesis and evaluation [5,6]. Peer collaboration further enriches the learning process by fostering dynamic interactions, challenging assumptions, and supporting co-construction of knowledge. This approach addresses gaps like the lack of real-time adaptability [9] and the underexplored role of AI in facilitating peer-driven creativity [13]. By linking these elements, the study advances theoretical understanding and offers practical insights for designing learner-centered educational environments. The findings demonstrate how ChatGPT can support higher-order cognitive processes and foster creativity, bridging individual and collaborative learning in scalable and impactful ways [2,10].

### 2.2. The study’s focus area

This research explores how technology-driven personalized learning (from ChatGPT), cognitive development through Bloom’s Taxonomy, and collaborative cognitive amplification work together (peer interaction) to foster creative thinking skills. ChatGPT plays a central role in personalized learning by providing adaptive feedback personalized to individual learning styles and needs. Bloom’s Taxonomy offers a structured pathway for guiding learners from basic knowledge to higher-order thinking, while peer interaction adds a collaborative layer that enhances creative problem-solving. By integrating these components, the study presents a cohesive framework for promoting creativity through the alignment of individual and group cognitive processes [5,6] (Fig 1).

**Fig 1:**
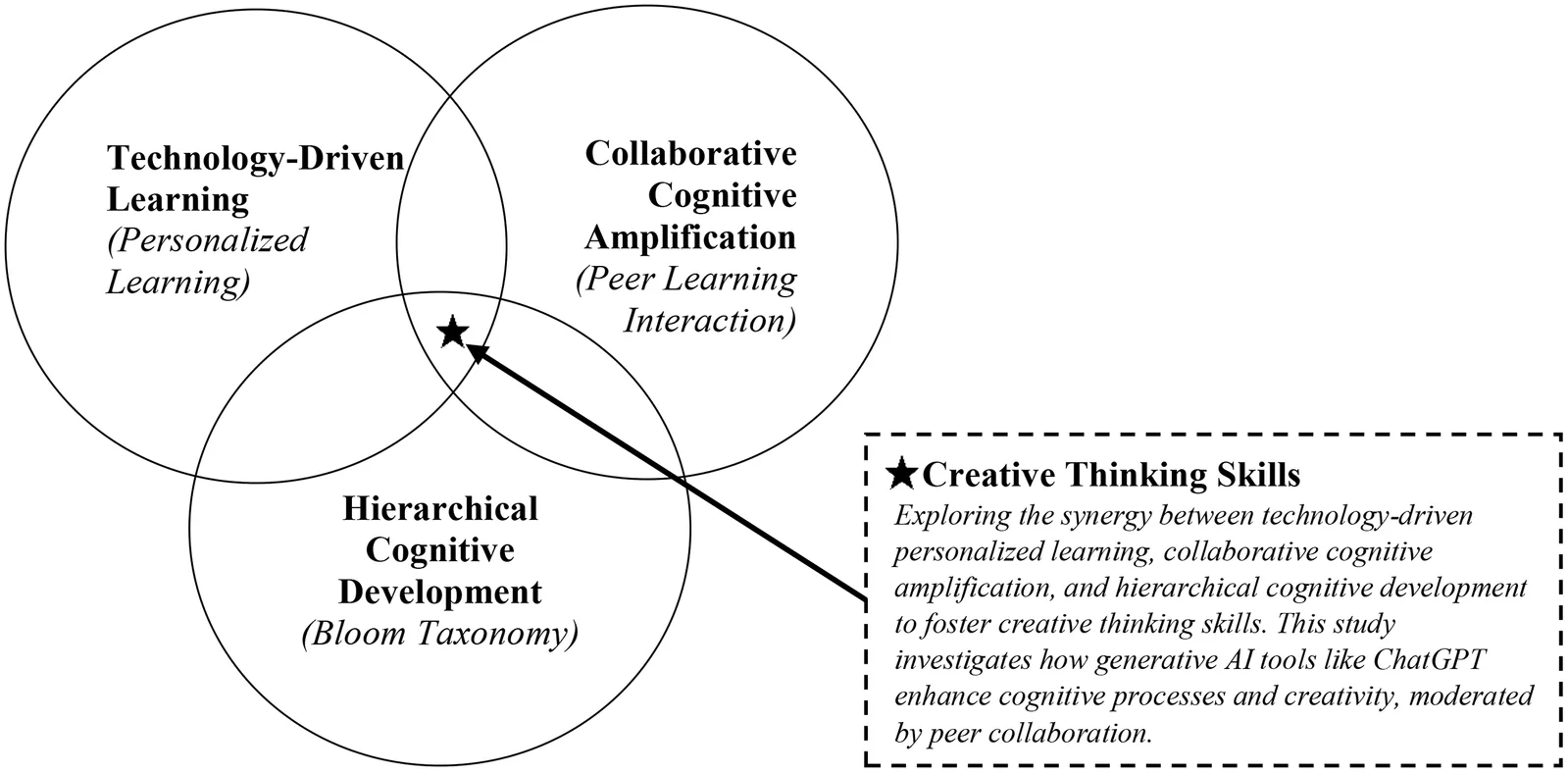
The study’s focus area.

ChatGPT enhances personalized learning by tailoring content and providing real-time, context-specific guidance. Through human-like interactions, it fosters engagement and critical inquiry, enabling learners to explore various perspectives and solutions. This approach supports Bloom’s hierarchical progression, where foundational knowledge and comprehension build toward higher-order skills like evaluation and creation [3,15]. However, over-reliance on AI tools can hinder the development of independent critical thinking. Including collaborative elements like peer interactions encourages reflective dialogue and knowledge co-construction, reducing the risk of passivity in learning environments [17].

Peer interactions in collaborative cognitive amplification bridge the gap between personalized learning and hierarchical cognitive development. Studies show that structured peer feedback and collaborative platforms enhance metacognitive awareness and drive innovative performance [23,24]. Combined with ChatGPT’s adaptive capabilities, peer interactions boost creative thinking by promoting diverse perspectives and iterative knowledge building. This approach helps learners tackle complex problems, integrate ideas from various disciplines, and develop innovative solutions. By aligning with Bloom’s higher-order cognitive dimensions, this dynamic learning model addresses the limitations of traditional teaching methods and provides educators with strategies for designing more effective and engaging learning environments [6,25].

This study combines personalized learning with ChatGPT, Bloom’s Taxonomy, and collaborative cognitive amplification to create a comprehensive framework for developing creative thinking skills. This approach enhances theoretical understanding and provides practical guidance for educators. It emphasizes balancing adaptive AI technologies with human-centered practices to ensure tools like ChatGPT support rather than replace traditional methods. By addressing cognitive and collaborative elements, the framework highlights AI’s potential to unlock creativity and transform learning across diverse educational contexts [10,14,23].

### 2.3. Personalized learning

Personalized learning through ChatGPT transforms education by creating adaptive learning pathways aligned with Bloom’s Taxonomy. It fosters progression from basic knowledge to creative thinking by providing personalized feedback and individualized content. Earlier systems, such as OntoPeFeGe, used Bloom’s framework to address learning gaps but lacked the adaptability ChatGPT offers through real-time guidance and interactive learning tasks [26]. ChatGPT supports critical reflection and deeper engagement by enabling learners to explore diverse problem-solving strategies and refine their understanding [8,16]. Unlike static cognitive graph systems, ChatGPT dynamically adjusts to student needs, promoting both cognitive progression and creative application in educational settings [27].

ChatGPT enhances personalized learning by advancing learners from analysis to creation through interactive prompts and tailored scenarios. It helps students critically evaluate, synthesize, and generate ideas while engaging in collaborative learning. Peer interactions supported by ChatGPT create opportunities for shared problem-solving and co-construction of knowledge, essential for fostering creativity [23,24]. AI-powered feedback systems, such as those highlighted in mobile learning studies, improve creative performance by enhancing reflection and metacognitive skills [16]. While challenges like over-reliance on AI and ethical concerns remain, ChatGPT bridges the gap between personalized learning and cognitive collaboration, offering an effective model for developing critical and creative thinking in education [26,27].

### 2.4. Bloom taxonomy

Bloom’s Taxonomy, originally developed to organize learning objectives, has become a foundation of modern education. Its structured approach guides cognitive development from basic recall to creative synthesis, helping educators foster higher-order thinking skills [28,29]. Anderson and Krathwohl’s [7] revision transformed the taxonomy from static categories to dynamic processes such as “remember” and “create,” integrating cognitive processes with knowledge dimensions. This update addressed contemporary educational needs by positioning creativity as the highest cognitive goal, encouraging alignment with complex learning tasks. Today, Bloom’s Taxonomy serves as both a theoretical framework and a practical tool for instructional design and assessment planning.

Integrating Bloom’s Taxonomy with artificial intelligence, particularly tools like ChatGPT, further enhances its relevance in modern learning environments. AI tools bring the taxonomy to life by delivering real-time, adaptive learning experiences personalized to individual needs. ChatGPT supports higher-order thinking by providing targeted feedback, simulating problem-solving scenarios, and fostering analytical engagement [16,30]. These systems also create personalized learning paths, enabling seamless progression through advanced tasks like evaluation and creation [8]. However, this dynamic interaction challenges the taxonomy’s linear structure, as learners often engage in iterative and nonlinear cognitive processes. This evolution calls for a reinterpretation of Bloom’s Taxonomy within AI ecosystems, shifting from static hierarchies to flexible, interconnected pathways.

AI integration has also expanded the domain-specific application of Bloom’s Taxonomy, particularly in areas requiring critical and creative problem-solving. For example, in programming education, tools like ChatGPT not only classify learning objectives but also enhance them by fostering collaboration and addressing skill gaps [31]. AI-driven assessment design further improves the alignment with cognitive goals by enhancing reliability and depth. Despite these advancements, caution is needed. Over-reliance on AI risks undermining independent critical thinking, a fundamental principle of Bloom’s framework, and raises ethical concerns about data privacy and equitable access [27]. As AI reshapes education, Bloom’s Taxonomy must evolve to support creativity and critical engagement in AI-enhanced environments.

The convergence of Bloom’s Taxonomy and AI, especially tools like ChatGPT, reveals untapped potential for transforming education. Unlike traditional methods with static progression, AI operationalizes the taxonomy by providing real-time, adaptive learning aligned with individual cognitive needs. ChatGPT enhances higher-order thinking by delivering targeted feedback and fostering analytical engagement, directly supporting the taxonomy’s goals [16,30]. While these advancements challenge the taxonomy’s linear framework, they also redefine how creativity and critical thinking are cultivated in education. By integrating Bloom’s Taxonomy with AI-driven systems, educators can create dynamic learning environments that promote flexible, innovative, and impactful learning experiences.

### 2.5. Creative thinking skills

The integration of Artificial Intelligence in education through tools like ChatGPT has transformed the development of creative thinking skills. Generative AI, including ChatGPT, uses extensive datasets and predictive capabilities to support creativity in unique ways. It enables learners to produce innovative outputs, simulate creative processes, and interact with dynamic content. This adaptability provides opportunities for solving problems, generating ideas, and fostering collaboration across disciplines [32,33]. In this study, ChatGPT is examined as a tool to cultivate creativity within the frameworks of Bloom’s Taxonomy and personalized learning. Its role in progressing cognitive skills aligns directly with the goal of enhancing creative thinking in educational environments [34].

Generative AI’s capacity to enhance creativity is evident across diverse educational contexts. ChatGPT facilitates interactive learning by enabling students to ask questions, explore perspectives, and evaluate alternatives, which supports creative problem-solving [16,34]. In engineering and arts education, ChatGPT aids in generating and critiquing outputs, providing a platform for experimentation and innovative exploration [33,35]. Computational creativity, exemplified by systems like IBM Watson, further highlights AI’s ability to recombine existing knowledge into novel solutions, inspiring students to refine their ideas [33]. This study focuses on how ChatGPT fosters creativity by bridging knowledge gaps, encouraging exploration, and enhancing the ability to synthesize and create new concepts [8].

The application of AI to enhance creative thinking must address ethical concerns and ensure that AI augments human creativity rather than replacing it. Misuse of AI, including plagiarism and over-reliance, risks diminishing critical and original thinking [36]. Educators must design learning environments where ChatGPT complements human creativity, allowing learners to maximize their potential through collaborative engagement [33]. By positioning ChatGPT as a partner in learning, this study highlights its role in supporting innovative thinking while promoting ethical and independent use. With its ability to foster exploration and creativity, AI becomes a transformative tool in achieving the educational objectives outlined in this study.

### 2.6. Theoretical framework

The conceptual framework of this study, presented in Fig 2, integrates ChatGPT-driven personalized learning, Bloom’s Taxonomy, and collaborative cognitive amplification through peer interaction to foster creative thinking skills. These elements are not isolated but dynamically interact to address both individual and collective learning processes. At the core of the framework lies the potential of ChatGPT as a personalized learning tool. By personalizing content and providing real-time, adaptive feedback, ChatGPT facilitates learners’ progression through Bloom’s Taxonomy, enabling deeper engagement with knowledge and cognitive tasks. This adaptive personalization aligns learning materials with students’ unique needs, encouraging the mastery of foundational knowledge while simultaneously setting the stage for higher-order cognitive processes like evaluation and creation [1,6].

**Fig 2:**
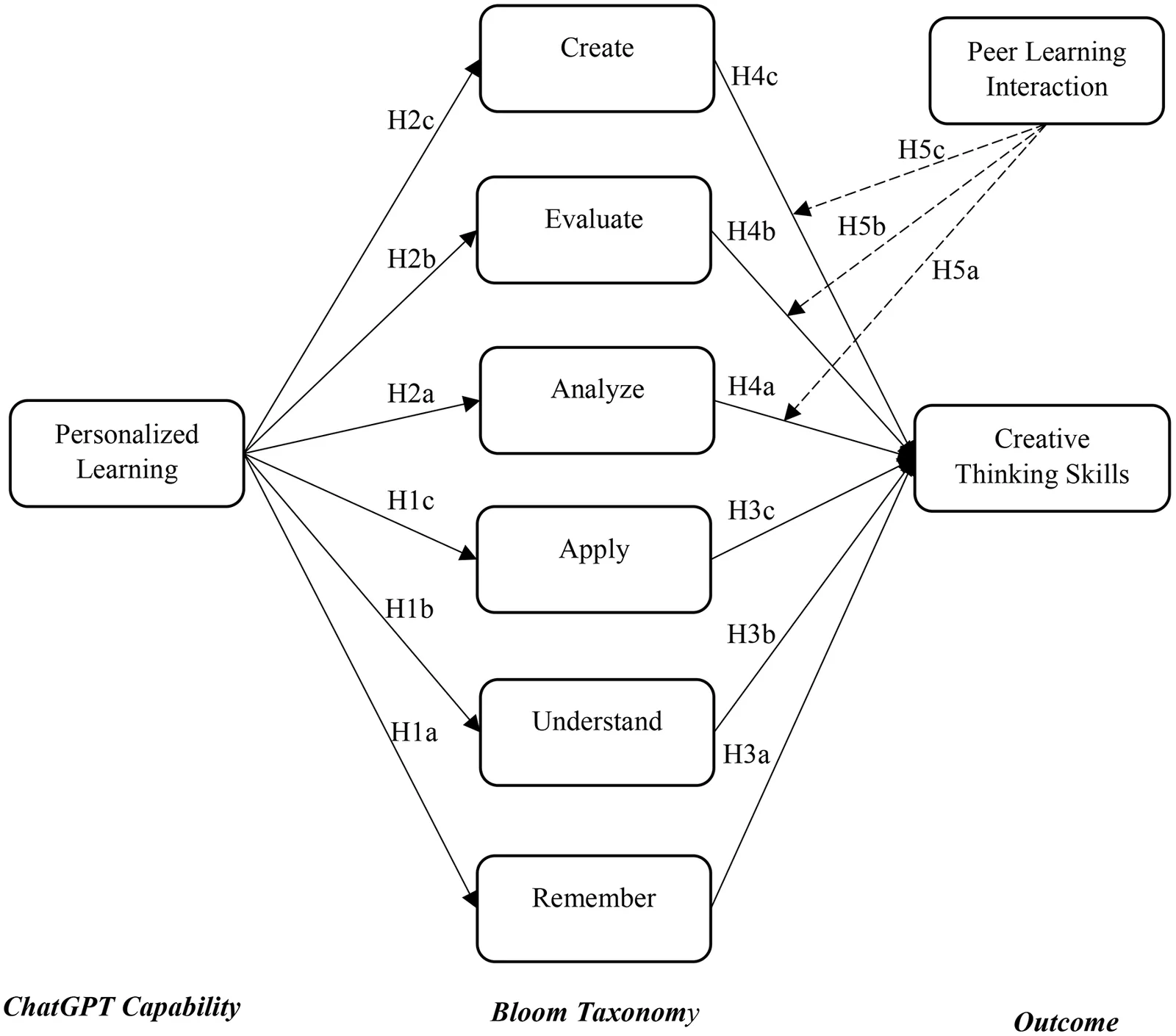
This study’s theoretical framework.

Bloom’s Taxonomy organizes cognitive tasks into hierarchical levels: remember, understand, apply, analyze, evaluate, and create. Integrating ChatGPT transforms this static framework into a dynamic and interactive process. At the foundational levels, ChatGPT enhances comprehension by offering explanations tailored to the learner’s current understanding. As learners advance, it facilitates application and analysis through scenario-based learning, where students solve problems with real-world relevance. At the highest levels of the taxonomy, ChatGPT supports evaluative reasoning and creative synthesis by presenting diverse perspectives, guiding iterative problem-solving, and encouraging innovation. This structured, progressive pathway ensures learners not only acquire cognitive depth but also cultivate the creative thinking necessary for solving complex challenges [14,16].

Collaborative cognitive amplification, through peer interaction, bridges personalized learning and collective knowledge construction. By fostering reflective dialogue and shared problem-solving, peer collaboration enhances creative thinking by exposing learners to diverse perspectives and promoting co-construction of ideas [2]. ChatGPT’s mediation in these collaborative environments acts as a substance, enriching group discussions with alternative viewpoints and guiding learners toward innovative solutions [24]. The interaction between personalized learning and peer collaboration creates a synergistic model, combining the depth of individual cognitive growth with the breadth of collaborative creativity [10]. This framework highlights how AI-driven tools like ChatGPT can transform learning environments into spaces that not only enhance cognitive progression but also equip learners with the creative capabilities required to thrive in complex, multidisciplinary contexts.

## 3. Hypothesis

### 3.1. Personalized learning and lower order thinking

ChatGPT’s personalized learning capabilities address foundational cognitive skills by enabling efficient information retrieval and reinforcing memory retention. It provides learners with instant access to tailored information, strengthening their ability to recall facts and foundational concepts with precision [2,5]. For example, ChatGPT generates customized quizzes and repetition-based exercises that support memory retention through iterative reinforcement [26,37]. Unlike traditional rote memorization techniques, ChatGPT adapts to individual learning needs, offering dynamic feedback that enhances the “remember” phase within Bloom’s Taxonomy. This adaptability positions ChatGPT as an essential tool for foundational learning, advancing beyond static educational methods [16]. Thus:

*H1a: Personalized learning from ChatGPT will significantly enhance the lower-order thinking ability of “remember.”*

Understanding involves interpreting and translating information, which ChatGPT facilitates by simplifying complex concepts through adaptive interaction. Its natural language processing capabilities allow students to ask questions and receive personalized clarifications, fostering deeper engagement with the material [3]. By encouraging learners to reframe concepts or compare perspectives, ChatGPT supports active learning, a critical aspect of the “understand” phase in Bloom’s Taxonomy [10]. Interactive dialogues promote self-explanation, a strategy proven to enhance comprehension and retention [6,14]. The integration of ChatGPT ensures learners transition from knowledge acquisition to meaningful understanding, addressing gaps in traditional teaching approaches. Therefore, this study proposes:

*H1b: Personalized learning from ChatGPT will significantly enhance the lower-order thinking ability of “understand.”*

The “apply” stage in Bloom’s Taxonomy focuses on using acquired knowledge in practical scenarios. ChatGPT supports this phase by simulating real-world problems and providing context-specific examples that bridge theoretical knowledge and practical application [11,33]. For instance, ChatGPT generates scenario-based exercises that challenge learners to solve problems, fostering active cognitive engagement [5]. By offering step-by-step guidance during these tasks, ChatGPT ensures learners gain confidence in applying knowledge to real-world challenges [8]. While AI tools like ChatGPT provide immense potential for enhancing application-based learning, careful integration into curricula is necessary to avoid over-reliance, which could limit the development of independent critical thinking skills [2]. Thus, we anticipated the following:

*H1c: Personalized learning from ChatGPT will significantly enhance the lower-order thinking ability of “apply”.*

### 3.2. Personalized learning and higher order thinking

The “analyze” level of Bloom’s Taxonomy emphasizes the ability to break down a complex information into components and understand relationships between them. ChatGPT facilitates analytical thinking by generating context-specific explanations and encouraging students to compare, contrast, and evaluate diverse perspectives [38]. For instance, scenario-based prompts provided by ChatGPT engage students in dissecting complex problems, enabling them to identify patterns and underlying structures [39]. Moreover, its adaptive feedback system allows students to refine their reasoning, fostering deeper engagement with analytical tasks [17]. Unlike traditional methods, ChatGPT’s capacity for personalized interactions enables students to revisit unclear areas, ensuring they achieve a more profound analytical understanding. Therefore, we propose the following:

*H2a: Personalized learning from ChatGPT will significantly enhance the higher-order thinking ability of “analyze”.*

Evaluative skills involve assessing information critically and making reasoned judgments, which ChatGPT supports through dynamic dialogue and feedback. By presenting arguments for and against a topic, ChatGPT helps students weigh evidence, prioritize criteria, and make informed decisions [38]. It aligns with constructivist learning theories by fostering reflective dialogue, allowing learners to interrogate and justify their conclusions. ChatGPT also facilitates evaluative thinking by simulating peer-review scenarios, challenging students to critique solutions or arguments effectively. While these features support evaluative thinking, they must be complemented by activities requiring independent judgment to prevent cognitive passivity and encourage critical engagement [2]. Thus:

*H2a: Personalized learning from ChatGPT will significantly enhance the higher-order thinking ability of “evaluate”.*

The “create” level represents the highpoint of Bloom’s Taxonomy, requiring students to synthesize knowledge and generate innovative ideas. ChatGPT promotes creativity by offering personalized prompts and open-ended questions that encourage learners to explore novel ideas and solutions [39]. Its ability to simulate real-world scenarios provides a platform for brainstorming and prototyping, especially in fields requiring inventive problem-solving, such as medical training or engineering design [17]. Furthermore, ChatGPT enhances this cognitive domain by iteratively refining student-generated outputs, ensuring originality and depth. Therefore:

*H2c: Personalized learning from ChatGPT will significantly enhance the higher-order thinking ability of “create”.*

### 3.3. Bloom taxonomy and creative thinking skills

Remembering, the foundational level of Bloom’s Taxonomy, provides the raw material for creativity by enabling the recall of essential facts and concepts. This cognitive process ensures that learners can access prior knowledge, which is critical for recombining information in novel and innovative ways [40,41]. In the context of ChatGPT, the ability to recall vast datasets allows it to present diverse and contextually relevant information, fostering creativity by inspiring users to draw connections across domains. For instance, a prompt requiring learners to retrieve facts about historical events can serve as a basis for developing creative narratives or exploring alternative scenarios. However, over-reliance on recall tasks alone may limit the scope for deeper engagement. This highlights the importance of integrating these tasks with higher cognitive processes for sustained creative growth [14]. We therefore propose the following:

*H3a: Remembering Through ChatGPT Will Enhance Creative Thinking Skills*

Understanding builds on the ability to remember by fostering comprehension, which is crucial for recognizing patterns and making meaningful connections between ideas. This deeper engagement forms the foundation of creative thinking, as it enables learners to see relationships between disparate pieces of information and conceptualize innovative solutions [42]. ChatGPT enhances this process by interpreting user inputs and generating coherent responses that promote reflection and comprehension. For example, when learners ask ChatGPT to explain a complex concept in simpler terms, the AI’s response can clarify underlying principles, allowing users to explore these ideas from new perspectives. While understanding facilitates creative ideation, ensuring balanced interaction with the AI is necessary to prevent students from becoming overly dependent on its interpretative capabilities [2]. Therefore:

*H3b: Understanding Through ChatGPT Will Enhance Creative Thinking Skills*

The “apply” level bridges theoretical knowledge and practical use which enable learners to test ideas in real-world contexts and foster innovative outcomes. In the ChatGPT context, tasks requiring the application of learned concepts, such as solving case studies or generating creative outputs, encourage users to experiment and refine their problem-solving approaches [11,43]. This adaptive interaction not only supports the synthesis of knowledge but also strengthens creativity through iterative exploration. For example, when prompted to apply a scientific theory to address a real-world challenge, ChatGPT facilitates the brainstorming of diverse solutions, empowering users to experiment with novel approaches. However, careful moderation is needed to ensure that these applications extend beyond formulaic responses to nurture genuine creative thought rather than mere AI-guided outputs. Therefore:

*H3c: Applying Through ChatGPT Will Enhance Creative Thinking Skills*

The cognitive process of analyzing involves breaking down information into its components to uncover patterns and relationships, making it essential for fostering creativity. ChatGPT supports this by enabling learners to engage with information in dynamic ways, presenting multiple perspectives and simplifying complex ideas to reveal new connections [2,44]. For instance, students analyzing AI-generated responses can uncover insights that fuel innovative thinking and encourage cognitive flexibility. Research by Zaidi et al. [44] emphasizes that fostering analytical reasoning promotes creative problem-solving, as it enables learners to challenge assumptions and explore hidden patterns. By facilitating iterative exploration and critical examination, ChatGPT aligns with the analytical processes that underpin creative thought, offering learners opportunities to uncover unique solutions. Thus:

*H4a: Analyzing Through ChatGPT Will Enhance Creative Thinking Skills*

Evaluating involves assessing ideas against established criteria to refine thought processes, a key step in developing advanced creativity. ChatGPT enhances evaluation by providing iterative feedback, generating alternative perspectives, and helping learners assess the relevance and originality of their ideas. As demonstrated by Ramirez [29] and Nagarajan and Sen [17], critical evaluation fosters deeper engagement with concepts, empowering learners to refine and enhance their creative output. For example, students can use ChatGPT to weigh the merits of AI-generated suggestions, improving their ability to distinguish viable ideas from less effective ones. This reflective process strengthens creativity by promoting critical judgment and enabling learners to iteratively refine their ideas into more impactful and innovative solutions. Therefore, we propose:

*H4b: Evaluating Through ChatGPT Will Enhance Creative Thinking Skills*

The process of creating, which synthesizes information to generate novel ideas, represents the highest cognitive level in Bloom’s Taxonomy and is inherently tied to creativity. ChatGPT serves as a powerful tool for stimulating creative thought by providing a platform for brainstorming, experimenting with diverse inputs, and iterating on ideas [10,45]. For example, students can use ChatGPT to simulate collaborative environments, where they build on generated content to develop unique projects and solutions. Research by Gaggioli [33] and Putra et al. [2] underscores that engaging in such creative processes equips learners with the skills to integrate diverse knowledge and approach problems with originality. ChatGPT enhances this by dynamically adapting to user needs, enabling learners to experiment with creative possibilities and bridge the gap between theoretical understanding and innovative application. We therefore propose the following:

*H4c: Creating Through ChatGPT Will Enhance Creative Thinking Skills*

### 3.4. Moderating effect of peer learning interaction

Peer learning interaction is a process where students work together to share ideas, critique each other’s thoughts, and solve problems collaboratively. This interaction encourages critical discussions, deeper understanding, and innovative thinking [46,47]. In the context of ChatGPT, peer interactions allow students to assess the content generated by AI critically. For example, ChatGPT can provide a foundation of ideas or scenarios, and peers can evaluate and refine these collaboratively, resulting in more creative and diverse outputs [2,48]. However, effective peer learning depends on positive, structured interactions. Poor-quality peer discussions can hinder progress, making it crucial to foster collaborative and supportive environments [49].

The process of analyzing involves breaking down complex ideas into smaller components, which lays the groundwork for creativity by identifying patterns and connections. Peer learning enhances this process by enabling learners to collaboratively dissect and critique information, uncovering insights that may not emerge in solitary learning [44,50]. For instance, when ChatGPT generates content to analyze, peers can collaboratively identify gaps, challenge assumptions, and explore alternative interpretations, thus deepening their analytical and creative thinking. This interaction not only enhances cognitive engagement but also encourages the synthesis of diverse ideas, ultimately leading to more innovative solutions. Therefore, when peer learning interaction will bridge the gaps between AI outputs and human creativity to ensure learners critically engage with the material rather than passively accepting it [51]. Thus:

*H5a: Peer Learning Interaction Moderates the Relationship Between Analyzing and Creative Thinking Skills*

Evaluation requires learners to assess the value and validity of information, an essential cognitive skill for fostering creativity. Peer learning enhances this process by creating opportunities for learners to challenge ideas, provide constructive feedback, and collectively refine their judgments. Unlike individual evaluation, where biases can dominate, peer interaction introduces diverse perspectives, encouraging a deeper exploration of creative possibilities [29,52]. For instance, ChatGPT may provide a range of solutions to a problem. Peers collaboratively critique these outputs, weighing their relevance and practicality to improve upon the ideas generated. This shared evaluation process enriches the overall creative outcome by combining AI-generated insights with human collaboration, creating a balanced approach to developing innovative solutions. The interplay between ChatGPT’s outputs and peer discussions highlights how collective assessment amplifies creative thinking by ensuring that ideas are both viable and novel [2,17]. Therefore, we hypothesize the following:

*H5b: Peer Learning Interaction Moderates the Relationship Between Evaluating and Creative Thinking Skills*

The creation process, which involves synthesizing information to generate new ideas, benefits significantly from peer learning interactions. Collaborative environments foster creativity by enabling learners to brainstorm, build on each other’s ideas, and experiment with different approaches [45]. ChatGPT can act as a facilitator by offering initial suggestions or frameworks, while peers refine and expand these ideas to suit their unique contexts and objectives. Unlike individual efforts, peer collaboration encourages iterative development, where ideas evolve through group input and constructive criticism. For example, in a group project, ChatGPT might provide a creative starting point, such as a concept for a design, which peers adapt and innovate upon to develop a more comprehensive and creative final product. This collective refinement ensures that the creative process benefits from multiple perspectives and critical engagement, bridging the strengths of AI with the dynamic nature of human collaboration [33,53]. Therefore, we propose the following:

*H5c: Peer Learning Interaction Moderates the Relationship Between Creating and Creative Thinking Skills*

## 4. Methods

### 4.1. Research design and process

This study explored the challenges of using ChatGPT for personalized learning, particularly in fostering higher-order cognitive skills like creativity within Bloom’s Taxonomy. Observations and informal interviews with educators and students revealed key concerns. While many found ChatGPT’s personalized feedback useful for idea generation, some noted that its responses lacked depth and did not fully support advanced cognitive processes such as synthesis and evaluation. Another issue was the risk of over-reliance on AI, which could hinder independent thought and creativity. Additionally, students and educators emphasized the role of peer collaboration in achieving deeper learning outcomes. These findings highlight the need to integrate collaborative learning into AI-driven educational models to enhance creative thinking skills.

Following the identification of these challenges, a comprehensive literature review was conducted to bridge the observed practical gaps with existing theoretical insights. Databases such as Google Scholar, Scopus, and WoS were utilized to explore studies on personalized learning, AI in education, and collaborative learning within Bloom’s Taxonomy. This review revealed theoretical gaps. For instance, while prior studies extensively explored the role of generative AI in enhancing personalized learning, they often overlooked its alignment with structured cognitive models like Bloom’s Taxonomy. Research by Zhang et al. [38] highlighted ChatGPT’s potential for knowledge dissemination but did not explore how its feedback mechanisms foster creativity or higher-order thinking. Similarly, Van Tran et al. [54] examined AI’s role in personalized education but did not investigate the moderating effects of peer collaboration in improving cognitive outcomes. These gaps were instrumental in shaping the central research questions of this study.

To bridge these gaps, this study examines how ChatGPT-driven personalized learning fosters higher-order cognitive skills, particularly creativity, within Bloom’s Taxonomy. It also explores the role of peer collaboration in enhancing AI-driven learning by promoting diverse perspectives and problem-solving. Understanding this interaction is crucial for optimizing cognitive outcomes in personalized education. By addressing these dimensions, this study aims to expand theoretical frameworks and provide practical insights for designing AI-enhanced learning environments that effectively integrate personalization and collaboration to support deeper cognitive development.

### 4.2. Operationalization and measures

Operationalization and measures of each construct were provided on Appendix Table A (S1 File).

#### 4.2.1. Personalized learning.

Personalized learning tailors education to individual learner needs, preferences, and goals [55,56]. Zhang et al. [38] highlight its core elements: goal alignment, adaptable resources, progress tracking, learner variability support, and exploratory engagement. In this study, personalized learning is assessed through five measures that evaluate ChatGPT’s ability to align with user goals, provide diverse materials, monitor progress, support unique needs, and foster exploration. These measures capture ChatGPT’s potential to enhance engagement and performance in personalized educational setting.

#### 4.2.2. Bloom taxonomy.

This study utilizes the revised Bloom’s taxonomy by Krathwohl [57], which serves as a hierarchical framework for understanding essential cognitive processes in learning. It focuses on six key dimensions: Remember, Understand, Apply, Analyze, Evaluate and Create. Within the context of ChatGPT-supported learning, this framework offers a structured approach to evaluate how foundational skills and higher-order thinking contribute to the development of creative thinking.

The *Remember* dimension focuses on recognizing and recalling key information, foundational for all higher-order cognitive processes [57]. To operationalize this construct, two measures are utilized: one assessing recognition of key concepts and the other examining the retrieval of specific information with ChatGPT’s assistance. These items ensure a detailed understanding of how foundational memory processes influence subsequent cognitive engagement.

*Understanding*, defined as the ability to grasp the meaning of information through processes like interpreting, exemplifying, and summarizing, is essential for deeper cognitive engagement [57]. This study employs six measures for this construct to capture diverse dimensions of understanding. These include interpreting ChatGPT outputs, using examples to clarify abstract ideas, and summarizing key points from interactions, among others. This broad set of measures ensures the comprehensive evaluation of learners’ ability to contextualize and internalize knowledge.

*Apply*, involves using knowledge to address real-world scenarios or execute tasks to bridge theory and practice [57]. The study uses two measures to evaluate this cognitive process, focusing on executing known methods with ChatGPT and applying insights to novel and unfamiliar contexts. These measures highlight the practical role of AI in enhancing learners’ ability to transfer theoretical knowledge to practical outcomes.

*Analysis* involves breaking down complex information into components and examining relationships between ideas [57]. Three measures capture this dimension, including differentiating between key components, organizing information into coherent structures, and identifying underlying causes of problems with ChatGPT’s support. These items provide a detailed view of how analytical processes influence creative problem-solving.

*Evaluation* requires learners to make judgments based on criteria or standards, a critical skill for reflective and independent thinking [57]. The study employs two measures for this construct, one assessing the ability to verify the accuracy of solutions and another examining the critique of proposed ideas.

*Creation* represents the highest cognitive level, focusing on synthesizing information to generate new ideas, solutions, or frameworks [57]. To operationalize this construct, three measures are used: generating innovative ideas, planning effective strategies, and producing original outputs.

#### 4.2.3. Creative thinking skills.

As defined by Sternberg et al. [58], creative thinking skills refer to the ability to generate novel, practical, and original ideas or solutions, which are crucial for innovation and complex problem-solving. In this study, these skills are operationalized within the context of AI-supported learning, focusing on how ChatGPT facilitates the production of creative outcomes. Drawing from Akpur [4] and Sternberg et al. [58], the measures include leveraging ChatGPT to inspire original ideas, adapt solutions to unique challenges, and explore innovative methods for academic and practical tasks. For instance, participants are assessed on their ability to use ChatGPT for brainstorming, demonstrating originality in group activities, and creating unique frameworks. This operationalization reflects the dynamic interaction between users and AI tools, emphasizing how ChatGPT supports fluency, flexibility, and originality in cognitive and creative processes [59,60].

#### 4.2.4. Peer learning interaction.

Peer learning interaction refers to collaborative engagements where individuals actively exchange ideas, perspectives, and support to achieve shared learning objectives. This process fosters communication, collaboration, and higher-order thinking skills, particularly in technology-mediated environments [61]. In this study, peer learning is conceptualized as the interplay between learners facilitated by ChatGPT, emphasizing collaboration, effective communication, and the division of tasks based on individual abilities. Measures include successful collaboration during ChatGPT-assisted learning, the provision of sufficient and useful information, and effective communication supported by ChatGPT’s tailored suggestions and feedback. These five measures underscore how ChatGPT enhances collaboration by enabling efficient task completion and dynamic group interactions, fostering deeper engagement and collective problem-solving [61,62].

### 4.2. Sampling technique and data collection procedure

This study uses an online survey to collect data from Indonesian students in higher education who have experience using ChatGPT for personalized learning. The main objective is to investigate how ChatGPT enhances personalized learning, bloom taxonomy (e.g., remember, understand, apply, analyze, evaluate and create) to achieve creative thinking skills. A non-probability, purposive sampling method is applied, with the following eligibility criteria for participants: 1) prior use of ChatGPT at least once for learning purposes; 2) current enrollment in a higher education institution; and 3) a minimum age of 18. These criteria were clearly outlined at the beginning of the questionnaire to ensure that only eligible participants contributed to the data collection.

The survey instrument consisted of four sections. The first section introduced the study and secured informed consent. The second section ensured participant eligibility based on predefined criteria. The third section gathered demographic information, including age, gender, education level, and ChatGPT usage patterns. The final section featured 28 items rated on a 7-point Likert scale, designed to measure constructs related to collaborative learning, cognitive engagement through Bloom’s Taxonomy, and critical thinking. Data collection occurred from 26^th^ November to 30^th^ January 2024, yielding 655 valid responses. To ensure validity and reliability, the questionnaire underwent expert review by two specialists in educational psychology and technology, followed by a pilot test with a small sample of students for refinement prior to distribution. This cross sectional survey study is reported in accordance with the Strengthening the Reporting of Observational Studies in Epidemiology (STROBE) guidelines.

### 4.3. Analysis technique

The research model and hypotheses are validated using Partial Least Squares Structural Equation Modeling with SmartPLS 4.0, following Hair et al.’s [63] guidelines for analyzing complex predictive models. A Common Method Variance (CMV) test, using Harman’s Single Factor Test, ensures no significant bias [64]. Convergent validity and reliability are confirmed through Average Variance Extracted (AVE) and Composite Reliability (CR) [63], while discriminant validity is assessed using the Fornell-Larcker criterion [65] and Heterotrait-Monotrait (HTMT) ratio [65]. The model’s explanatory power is measured by R² values, with f² effect sizes evaluating predictor contributions. Bootstrapping with 5,000 resamples tests path coefficient significance, ensuring robust and reliable results. This rigorous approach provides a comprehensive evaluation of ChatGPT’s role in advancing personalized learning, bloom taxonomy to achieve creative thinking skills.

## 5. Results

### 5.1. Sample profile

The study’s sample highlights a diverse and inclusive demographic as shown in Table 2. Gender representation is balanced, with males accounting for 54% and females 46%. Participants are mainly in the younger age brackets, with 76% between 18 and 35 years old, a group highly engaged with emerging technologies like ChatGPT. Marital status reveals that 69% of respondents are single, reflecting a population likely to be involved in education or early career development. Educational attainment shows that nearly half hold bachelor’s degrees, while 21% possess advanced or vocational qualifications, indicating a well-educated sample suitable for evaluating higher-order cognitive skills. The participants’ fields of study span technology, social sciences, education, design, and STEM. This reflects interdisciplinary insights into how AI tools impact cognitive and creative learning. The study’s demographic diversity enhances the study’s relevance and strengthens its capacity to produce well-rounded findings.

**Table 2:** Sample profile.

Measures	Criteria	F	P
Gender	Male	288	0.54
	Female	245	0.46
Age	18 - 25	155	0.29
	26 - 35	251	0.47
	36 - 45	101	0.19
	>45	27	0.05
Marital Status	Single	369	0.69
	Married	164	0.31
Educational Background	Senior High or Equivalent	165	0.31
	Diploma (Vocational Education)	64	0.12
	Bachelor Level	256	0.48
	Graduate Levels	48	0.09
Field of Study	Technology Information	69	0.13
	Education	117	0.22
	Psychology	112	0.21
	Social Sciences and Humanities	123	0.23
	Design	75	0.14
	STEM	26	0.05
	Economics	12	0.02

### 5.2. Common method variance

To address the potential concern of CMV in this study, Harman’s single-factor test and VIF analysis were conducted using SPSS. The single-factor test revealed eigenvalues ranging from 1.145 to 11.809, with the largest single factor explaining only 11% of the variance, well below the 50% threshold, indicating minimal CMV impact [64]. Additionally, VIF values ranged from 1.000 to 3.896, confirming no multicollinearity concerns among the variables. These combined results suggest that CMV does not pose a significant issue, ensuring the reliability and validity of the study’s data and findings.

### 5.3. Validity and reliability assessment

The study confirms the validity and reliability of its measurement model through convergent validity and internal consistency as shown in Table 3. Outer loadings for all items exceeded 0.70, showing strong item-to-construct relationships. Cronbach’s Alpha values ranged from 0.701 to 0.835, reflecting high internal consistency, while composite reliability values surpassed 0.832, ensuring robust reliability. The AVE values between 0.553 and 0.789 demonstrate that constructs capture sufficient variance. Constructs such as “Analyze” (AVE = 0.697, CR = 0.873) and “Creative Thinking Skills” (AVE = 0.594, CR = 0.880) met all thresholds for inclusion. These results validate the constructs’ suitability for the study and confirm that the measures provide a reliable foundation for the analysis [63].

**Table 3:** Convergent validity and reliability.

Constructs	Items	OL	α	CR	AVE
Analyze	AN.1	0.794	0.782	0.873	0.697
	AN.2	0.856			
	AN.3	0.853			
Apply	AP.1	0.875	0.734	0.882	0.789
	AP.2	0.902			
Create	CE.1	0.816	0.753	0.859	0.670
	CE.2	0.824			
	CE.3	0.814			
Creative Thinking Skills	CTS.1	0.774	0.829	0.880	0.594
	CTS.2	0.755			
	CTS.3	0.783			
	CTS.4	0.777			
	CTS.5	0.765			
Evaluate	EV.1	0.880	0.701	0.870	0.770
	EV.2	0.875			
Peer Learning Interaction	PLI.1	0.750	0.735	0.832	0.553
	PLI.2	0.704			
	PLI.3	0.750			
	PLI.4	0.768			
Personalized Learning	PL.1	0.713	0.809	0.867	0.566
	PL.2	0.733			
	PL.3	0.792			
	PL.4	0.767			
	PL.5	0.754			
Remember	RR.1	0.870	0.711	0.874	0.776
	RR.2	0.891			
Understand	UD.1	0.798	0.835	0.879	0.574
	UD.2	0.776			
	UD.3	0.732			
	UD.4	0.702			
	UD.5	0.787			
	UD.6	0.743			

Notes:

a. AN, Analyze; AP, Apply; CE, Create; CTS, Creative Thinking Skills; EV, Evaluate; PLI, Peer Learning Interaction; PL, Personalized Learning; RR, Remember; UD, Understand.

b. The threshold for OL, Outer Loadings > 0.70; α, Cronbach’s Alpha > 0.70; CR, Composite Reliability 0.70; AVE, Average Variance Extracted > 0.50.

This study assessed discriminant validity using the Fornell-Larcker Criterion and the HTMT method. According to the Fornell-Larcker approach, the square root of AVE values (diagonal bolded values) exceeded the inter-construct correlations across all constructs, affirming adequate discriminant validity [65]. For instance, the square root of AVE for Analyze (0.835) exceeded its correlations with other constructs, such as Apply (0.821) and Creative Thinking Skills (0.820). The HTMT analysis, recommended by Henseler et al. [66], further confirmed discriminant validity with values below the threshold of 0.85, indicating strong validity across constructs. For example, HTMT between Analyze and Apply was 0.821, which is within the acceptable range. Therefore, this study’s discriminant validity has robustness of the measurement model, as summarized in Table 4.

**Table 4:** Fornell-larcker criterion and HTMT.

	AN	AP	CE	CTS	EV	PLI	PL	RR	UD
AN	**0.835**	*0.821*	*0.602*	*0.820*	*0.857*	*0.053*	*0.671*	*0.828*	*0.842*
AP	0.623	**0.888**	*0.566*	*0.817*	*0.837*	*0.071*	*0.626*	*0.542*	*0.832*
CE	0.463	0.423	**0.818**	*0.723*	*0.739*	*0.200*	*0.804*	*0.792*	*0.543*
CTS	0.741	0.639	0.571	**0.771**	*0.823*	*0.134*	*0.775*	*0.672*	*0.849*
EV	0.635	0.600	0.537	0.705	**0.877**	*0.143*	*0.812*	*0.672*	*0.812*
PLI	−0.035	−0.053	−0.140	−0.107	−0.106	**0.743**	*0.194*	*0.154*	*0.072*
PL	0.542	0.488	0.869	0.638	0.615	−0.142	**0.752**	*0.854*	*0.626*
RR	0.622	0.394	0.580	0.518	0.477	−0.118	0.657	**0.881**	*0.554*
UD	0.682	0.655	0.438	0.727	0.702	−0.052	0.526	0.435	**0.741**

Notes:

a. AN, Analyze; AP, Apply; CE, Create; CTS, Creative Thinking Skills; EV, Evaluate; PLI, Peer Learning Interaction; PL, Personalized Learning; RR, Remember; UD, Understand.

b. The diagonal bolded values indicated the square root of AVE.

c. The italicized values indicate HTMT.

d. The threshold for HTMT < 0.85 strong, < 0.90 moderate and < 0.95 weak.

Discriminant validity was assessed using the cross-loadings matrix, comparing each item’s loading on its intended construct with its loadings on others. Results confirm that items load more strongly on their respective constructs than on others, meeting the criteria for construct distinction and supporting the validity of the measurement model [63]. The cross-loadings matrix presented in Table 5.

**Table 5:** Cross-loadings matrix.

	Analyze	Apply	Create	Creative Thinking Skills	Evaluate	Peer Learning Interaction	Personalized Learning	Remember	Understand
AN.1	**0.794**	0.502	0.340	0.624	0.530	−0.006	0.399	0.394	0.573
AN.2	**0.856**	0.497	0.391	0.613	0.489	−0.017	0.448	0.539	0.555
AN.3	**0.853**	0.558	0.426	0.620	0.570	−0.063	0.506	0.617	0.581
AP.1	0.553	**0.875**	0.343	0.542	0.523	−0.038	0.397	0.332	0.539
AP.2	0.554	**0.902**	0.406	0.592	0.544	−0.054	0.468	0.367	0.622
CE.1	0.354	0.365	**0.816**	0.472	0.411	−0.173	0.715	0.449	0.348
CE.2	0.400	0.348	**0.824**	0.444	0.422	−0.016	0.714	0.496	0.358
CE.3	0.383	0.326	**0.814**	0.487	0.485	−0.152	0.703	0.478	0.370
CTS.1	0.567	0.496	0.467	**0.774**	0.596	−0.100	0.499	0.404	0.546
CTS.2	0.554	0.511	0.449	**0.755**	0.486	−0.036	0.488	0.408	0.543
CTS.3	0.559	0.417	0.413	**0.783**	0.545	−0.069	0.487	0.364	0.552
CTS.4	0.568	0.546	0.460	**0.777**	0.497	−0.104	0.527	0.406	0.552
CTS.5	0.606	0.490	0.414	**0.765**	0.589	−0.099	0.461	0.411	0.607
EV.1	0.615	0.521	0.483	0.614	**0.880**	−0.084	0.556	0.448	0.667
EV.2	0.498	0.533	0.460	0.624	**0.875**	−0.103	0.522	0.388	0.564
PL.1	0.264	0.291	0.571	0.404	0.397	−0.155	**0.713**	0.337	0.313
PL.2	0.472	0.376	0.494	0.491	0.463	−0.084	**0.733**	0.558	0.446
PL.3	0.414	0.416	0.724	0.506	0.451	−0.152	**0.792**	0.502	0.401
PL.4	0.431	0.374	0.743	0.464	0.458	−0.014	**0.767**	0.532	0.391
PL.5	0.430	0.366	0.711	0.524	0.531	−0.142	**0.754**	0.511	0.413
PLI.1	−0.031	−0.059	−0.120	−0.087	−0.121	**0.750**	−0.118	−0.083	−0.053
PLI.3	−0.019	−0.037	−0.032	−0.053	−0.052	**0.704**	−0.030	−0.038	−0.012
PLI.4	−0.019	−0.042	−0.113	−0.078	−0.058	**0.750**	−0.123	−0.103	−0.039
PLI.5	−0.033	−0.020	−0.125	−0.090	−0.074	**0.768**	−0.125	−0.110	−0.040
RR.1	0.521	0.317	0.496	0.433	0.382	−0.102	0.557	**0.870**	0.351
RR.2	0.573	0.375	0.524	0.478	0.455	−0.106	0.598	**0.891**	0.414
UD.1	0.519	0.566	0.415	0.583	0.514	−0.023	0.487	0.371	**0.798**
UD.2	0.547	0.560	0.333	0.582	0.522	−0.081	0.394	0.392	**0.776**
UD.3	0.489	0.483	0.245	0.485	0.456	−0.013	0.323	0.273	**0.732**
UD.4	0.494	0.412	0.282	0.523	0.552	−0.025	0.346	0.264	**0.702**
UD.5	0.429	0.459	0.258	0.486	0.415	−0.037	0.293	0.229	**0.787**
UD.6	0.545	0.425	0.382	0.558	0.642	−0.048	0.456	0.374	**0.743**

### 5.4. Hypothesis testing

The results of hypothesis testing, outlined in Tables 6 and 7 and illustrated in Fig 3, provide critical evidence supporting the conceptual framework of this study. Personalized learning demonstrates a substantial influence on the cognitive processes within Bloom’s taxonomy, reflecting its foundational role in shaping cognitive engagement. Specifically, the relationships tested in H1a, H1b, and H1c confirm that personalized learning significantly enhances remembering (β = 0.657, T = 15.618), understanding (β = 0.526, T = 11.519), and applying (β = 0.488, T = 10.075), with all effects highly significant at ***p < 0.001. These findings emphasize the strength of personalized learning in equipping learners with the essential skills to process, comprehend, and utilize information effectively. Similarly, personalized learning positively drives higher-order thinking skills, including analyzing (H2a, β = 0.542), evaluating (H2b, β = 0.615), and creating (H2c, β = 0.869), confirming its pivotal role in facilitating deeper cognitive engagement.

**Table 6:** Summary of hypothesis testing.

Hypothesis	*β*	T-Value	Bootsrapping CI 97.5% N = 5000		Decision
			Min	Max	
H1a. PL → RR	0.657***	15.618	0.570	0.734	Supported
H1b. PL → UD	0.526***	11.519	0.435	0.614	Supported
H1c. PL → AP	0.488***	10.075	0.391	0.579	Supported
H2a. PL → AN	0.542***	10.189	0.428	0.640	Supported
H2b. PL → EV	0.615***	13.592	0.518	0.697	Supported
H2c. PL → CE	0.869***	30.512	0.808	0.919	Supported
H3a. RR → CTS	−0.009	0.227	−0.091	0.064	Not Supported
H3b. UD → CTS	0.223***	3.596	0.118	0.359	Supported
H3c. AP → CTS	0.095*	1.992	0.002	0.187	Supported
H4a. AN → CTS	0.336***	6.628	0.233	0.430	Supported
H4b. EV → CTS	0.175***	3.517	0.071	0.268	Supported
H4c. CE → CTS	0.172***	4.169	0.086	0.248	Supported

Notes:

a. AN, Analyze; AP, Apply; CE, Create; CTS, Creative Thinking Skills; EV, Evaluate; PLI, Peer Learning Interaction; PL, Personalized Learning; RR, Remember; UD, Understand.

b. *β,* Path Coefficient; CI, Confidence Interval.

c. Significance level *P < 0.05; ***P < 0.001.

**Table 7:** Summary of moderating effect.

Hypothesis	*β*	T-Value	Bootsrapping CI 97.5% N = 5000		Decision
			Min	Max	
H5a. PLI x AN → CTS	−0.061	1.298	−0.141	0.047	Not Supported
H5b. PLI x EV → CTS	−0.007	0.151	−0.097	0.087	Not Supported
H5c. PLI x CE → CTS	0.084*	2.066	0.004	0.165	Supported

Notes:

a. AN, Analyze; CE, Create; CTS, Creative Thinking Skills; EV, Evaluate; PLI, Peer Learning Interaction.

b. *β,* Path Coefficient; CI, Confidence Interval.

c. Significance level *P < 0.05.

**Fig 3:**
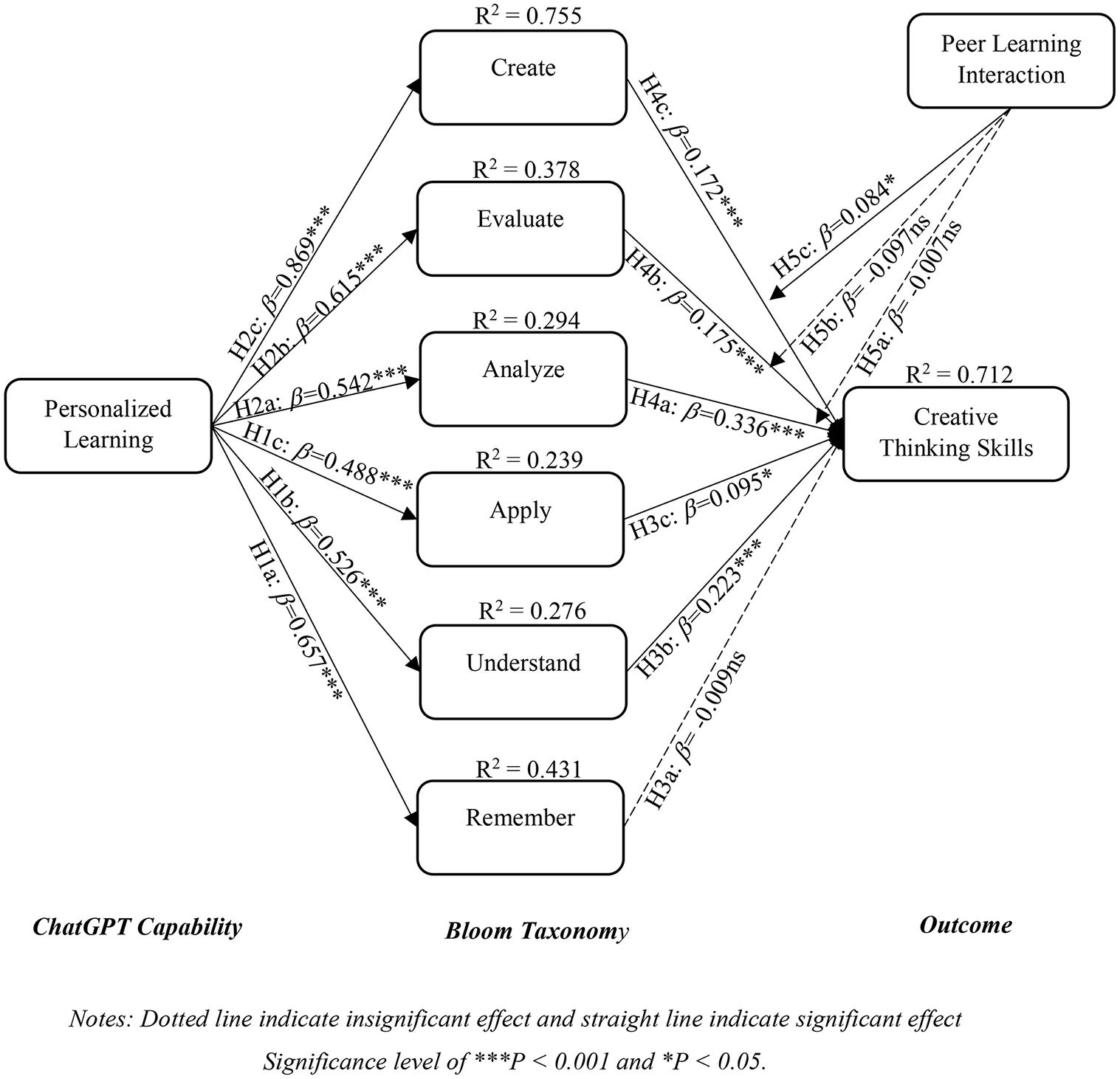
Summary of hypothesis testing.

The relationship between cognitive processes and creative thinking skills reveals important distinctions in their influence. While remembering (H3a) does not show a significant impact (β = −0.009, T = 0.227), understanding (H3b, β = 0.223, T = 3.596), applying (H3c, β = 0.095, T = 1.992), analyzing (H4a, β = 0.336, T = 6.628), evaluating (H4b, β = 0.175, T = 3.517), and creating (H4c, β = 0.172, T = 4.169) significantly enhance creative thinking skills. These findings emphasize that higher-order thinking processes, particularly analyzing and creating, are vital drivers of creativity. While lower-order skills like remembering provide foundational knowledge, it is the more complex cognitive processes that enable individuals to critically assess, adapt, and generate innovative solutions. This highlights the importance of fostering advanced cognitive engagement to enhance creativity in learning environments.

The moderating role of peer learning interaction is less uniform across the tested relationships. While the interaction between peer learning and analyzing (H5a, β = −0.061, T = 1.298) and evaluating (H5b, β = −0.007, T = 0.151) does not yield significant effects, its interaction with creating (H5c, β = 0.084, T = 2.066, *p < 0.05) provides compelling evidence of its selective importance. This moderating effect highlights that while peer learning interaction does not universally enhance the cognitive-to-creativity pathway, it is critical when paired with creative tasks, facilitating collaboration and idea generation. These results validate the theoretical assumptions of this study, where the complex interplay between personalized learning, cognitive processes, and peer dynamics drives creative outcomes.

### 5.5. Model robustness testing

The robustness of the model was evaluated using fit indices and R² values, both of which provided a comprehensive view of the model’s structural integrity and explanatory strength. The fit indices revealed acceptable results, with an SRMR of 0.065, NFI of 0.954, and chi-square (χ²) of 422.654, all adhering to recommended thresholds [63], thereby confirming the structural soundness of the model. Additional indices, such as d_ULS (0.262) and d_G (0.174), further validated the model’s fit [63]. The R² values highlighted the model’s ability to explain variance across key constructs. Specifically, Creative Thinking Skills exhibited an R² of 0.712, while Create recorded 0.755, both showcasing substantial explanatory power. Other constructs, such as Analyze (0.294), Apply (0.239), Evaluate (0.378), Remember (0.431), and Understand (0.276), exceeded the baseline threshold of 0.1, underscoring the model’s robustness.


$$\begin{array}{c} GoF=\sqrt{\overset{―}{AVE}\;x\;\overset{―}{R^{\text{2}}}} \end{array}$$ (Eq. 1)

$$\begin{array}{c} =\sqrt{\overset{―}{\text{0}.\text{6}\text{6}\text{5}}\;x\;\overset{―}{\text{0}.\text{4}\text{4}\text{1}}} \end{array}$$

= 0.541

The Goodness of Fit (GoF) index was calculated using the formula eq. 1, yielding a score of 0.541, which exceeds the high-fit threshold outlined by Tenenhaus et al. [67] and Wetzels et al. [68]. This score reflects the model’s strong structural fit and reliability, positioning it well within the criteria for robust models. These findings affirm the model’s capacity to effectively capture and explain the underlying relationships, reinforcing its applicability for investigating creative thinking skills and associated constructs.

Goodness of Fit.

## 6. Discussion

This study demonstrates the transformative impact of integrating personalized learning, Bloom’s Taxonomy, and peer learning interaction on fostering creative thinking skills. Personalized learning, mainly when supported by ChatGPT, is significantly associated with students’ cognitive engagement across Bloom’s processes, including remembering, understanding, applying, analyzing, evaluating, and creating. ChatGPT provides adaptive feedback personalized to individual needs, encouraging learners to deepen their engagement and apply knowledge creatively. This interpretation is consistent with recent evidence showing that ChatGPT can support personal learning environments by providing personalized resources, tailored feedback, metacognitive support, creativity support, and critical-thinking activities [69]. However, one notable drawback of this approach is the risk of over reliance on AI generated guidance, which may reduce learners’ independent evaluation of information, especially when tasks require originality and ownership. This highlights the importance of balancing AI tools with strategies that promote autonomous critical thinking. It is also plausible that differences in digital readiness and patterns of ChatGPT use condition these benefits, which should be examined in future research.

The findings reveal that personalized learning significantly strengthens both foundational and advanced cognitive skills. Rather than reflecting only faster content access, the results suggest that ChatGPT supported personalization is linked to perceived engagement in higher order processes such as analyzing, evaluating, and creating. This dynamic adaptability sets ChatGPT apart from traditional teaching methods, which often rely on static content delivery. For example, students can use ChatGPT to clarify assumptions, compare alternatives, and iteratively refine responses in scenario based tasks, which maps onto analyzing, evaluating, and creating. These findings extend Mogavi et al.’s [1] work by illustrating how ChatGPT related personalized learning aligns with specific higher order cognitive processes beyond basic comprehension. At the same time, research using Bloom’s Taxonomy to assess generative AI performance indicates that AI-supported learning and assessment require careful task design, because generative AI may perform unevenly across different cognitive levels [70]. Because the data are cross sectional and self reported, these interpretations describe associative patterns rather than causal effects.

Higher order cognitive processes, such as analysis and creation are essential for developing creative thinking. Analysis involves breaking down information, identifying patterns, and forming meaningful connections, which are key elements of innovation. Consistent with this logic, analyzing shows the strongest positive relationship with creative thinking skills, with creating also contributing positively, whereas remembering is not significant. These findings support Akpur’s [4] argument that higher order thinking is crucial for academic success. They are also consistent with evidence showing that generative AI applications can positively influence students’ innovation capability by strengthening critical thinking, creative problem solving, and adaptive learning processes [71]. The non significant association for remembering suggests that access to information alone, even when facilitated by AI, does not automatically translate into creativity without subsequent transformation through higher order processing. Because this study does not observe task performance directly, the mechanisms are inferred from the pattern of associations and should be tested using performance based designs.

Peer learning interaction played a key role in enhancing creative outcomes, especially in tasks requiring higher order thinking. Empirically, peer learning interaction significantly moderates the relationship between creating and creative thinking skills, while moderation is not significant for analyzing or evaluating. Structured peer discussions encouraged reflective dialogue and knowledge co construction, deepening learners’ engagement with complex problems. A plausible mechanism is that creation tasks are open ended and benefit from combining perspectives, negotiating novelty and usefulness, and iteratively revising drafts, processes that peers can supply beyond what AI produces. This selective moderation effect aligns with findings that equal and active participation in synchronous collaborative learning supports joint information processing and learning gains, particularly when learners engage in idea generation and knowledge creation [72]. In contrast, analyzing and evaluating may receive substantial scaffolding from ChatGPT through comparisons, counterarguments, and suggested criteria, which can reduce the incremental contribution of peer interaction in those pathways. In collaborative settings, facilitation remains important to ensure inclusive participation and to position ChatGPT as a stimulus for critique rather than a validator of final answers.

The results further highlight the impact of peer collaboration on creative problem solving. Collaborative environments enabled learners to integrate diverse perspectives and refine ideas, leading to innovative solutions. This interpretation is consistent with the significant moderation at the creation level and aligns with Wu et al. [24] on peer supported innovation in learning. A practical implication is that educators can design creation oriented activities where ChatGPT supports idea generation while peers evaluate originality, contextual fit, and feasibility, then revise outputs accordingly. However, unstructured collaboration sometimes led to inefficiencies, so educators should establish clear guidelines and objectives to keep discussions productive. Because peer learning interaction was measured as perceived collaborative effectiveness rather than discourse quality, future work should test whether argumentation quality and critique depth produce stronger moderation for analyzing and evaluating.

While ChatGPT fosters creativity and cognitive growth, over reliance on the tool presents a significant challenge. In the context of this study, the risk is most salient because personalized learning strongly predicts higher order processes, and creative thinking is most closely linked to analyzing and creating. Over reliance may manifest when students adopt AI generated arguments or ideas with limited interrogation, resulting in outputs that are fluent but generic, weakly contextualized, or insufficiently justified. This aligns with concerns raised by Putra et al. [2] about the risks of excessive AI reliance in education. The selective moderation result suggests a concrete mitigation pathway: during creation tasks, structured peer critique can pressure learners to justify choices, adapt AI suggestions, and demonstrate ownership, thereby supporting creativity while reducing uncritical adoption. This underscores the need for instructional strategies that promote reflective practices, helping learners critically assess and refine AI inputs. However, ChatGPT proved valuable as a facilitator rather than a replacement for human creativity. When used thoughtfully, it supported exploration and innovation by providing a foundation for idea development. Interpretation of these findings should also consider limitations related to validity. The purposive sampling approach and the online self report design may limit generalizability and introduce self selection. The cross sectional design constrains causal inference, and unmeasured factors such as AI literacy, intensity of use, or course assessment design may influence both peer interaction and creativity.

## 7. Implication

### 7.1. Implication for theory

This research deepens the theoretical understanding of how generative AI, particularly ChatGPT, transforms learning by integrating personalized learning, Bloom’s Taxonomy, and peer collaboration to enhance creative thinking. Unlike previous studies (e.g.[1,9]), which focused on static assessment models or isolated cognitive dimensions, this study highlights the dynamic interaction between AI-driven adaptability and cognitive progression. It extends Bloom’s Taxonomy by showing how ChatGPT enables simultaneous engagement across multiple cognitive levels, from foundational knowledge acquisition to higher-order processes like synthesis and evaluation. These challenges traditional linear cognitive frameworks, proposing a more flexible, iterative learning pathway driven by AI—an aspect largely overlooked in prior research.

This study advances personalized learning theory by demonstrating how ChatGPT’s adaptive capabilities provide tailored feedback that aligns with learners’ cognitive needs. Unlike static digital learning systems, ChatGPT adjusts its guidance in real time, bridging the gap between foundational learning and creative application. This finding addresses gaps identified by DeMara et al. [9], showing that personalized learning extends beyond cognitive scaffolding to support innovative problem-solving and higher-order thinking. However, the study also reveals the risks of over-reliance on AI, where learners become less autonomous in critical thinking. This underscores the need for frameworks that balance AI guidance with strategies that promote independent cognitive engagement.

Moreover, this study deepens the understanding of collaborative learning by illustrating how ChatGPT enhances peer interactions in fostering both knowledge construction and creative problem-solving. By integrating ChatGPT into discussions, learners engage with diverse perspectives, critically assess ideas, and refine their thinking through collective input. This expands on Lijie et al. [13] by shifting the focus from individual learning to a collaborative setting, where AI acts as a facilitator rather than a passive tool. However, the research also highlights challenges such as dominant voices overshadowing quieter participants and inefficiencies in unstructured discussions. These insights emphasize the need for guided facilitation to ensure balanced participation and productive collaboration. Ultimately, the findings suggest that while ChatGPT enriches group creativity, its role should complement rather than replace human-driven interaction.

Finally, this research advances the theoretical discourse on creative thinking by integrating cognitive flexibility theory into AI-driven learning. The findings show that ChatGPT fosters reflective and analytical reasoning by exposing learners to diverse perspectives and evaluative challenges, promoting the synthesis of novel ideas. This aligns with the theoretical foundations of creativity while addressing gaps in prior research, such as those noted by Akpur [4] and Putra et al. [2], which underestimated AI’s role in supporting iterative, interdisciplinary knowledge building. Beyond redefining cognitive progression in the AI era, this study offers practical insights for designing adaptive, inclusive, and innovation-driven educational frameworks.

### 7.2. Implication for practice

This study demonstrates the pivotal role of personalized learning in enhancing both cognitive engagement and creative thinking, establishing it as an essential framework for contemporary education. Personalized learning personalizes educational experiences to meet the diverse needs of students, fostering their ability to process, apply, and create knowledge effectively. For instance, adaptive learning platforms, supported by AI tools like ChatGPT, can dynamically adjust instructional content based on individual progress and preferences. Such approaches not only empower learners to engage meaningfully with complex concepts but also encourage autonomous learning and self-efficacy. Previous studies have highlighted the effectiveness of personalized learning in boosting comprehension and application in varied contexts [73,74].

Higher-order cognitive processes, including analyzing, evaluating, and creating, are critical drivers of creative thinking, as demonstrated in this research. While foundational skills such as remembering and understanding lay the groundwork for knowledge acquisition, it is advanced cognitive engagement that enables learners to critically assess information and produce innovative solutions. For example, structured activities that require students to analyze case studies or evaluate real-world scenarios promote deeper cognitive processing and enhance creativity. Educators should integrate tasks that necessitate these advanced skills, such as collaborative design projects or critical debates. Studies by de Vicente-Yagüe-Jara et al. [75] and Maghsudi et al. [76] affirm that engaging learners in higher-order thinking fosters creativity and equips them for complex problem-solving.

Peer learning interaction plays a nuanced role in enhancing creative outcomes, particularly in collaborative and idea-generation tasks. While its impact may not uniformly extend to all cognitive processes, peer interaction is essential for fostering creativity when students work together on challenging, innovative activities. For example, integrating peer-led brainstorming sessions in group projects can stimulate diverse perspectives and co-create novel solutions. AI tools like ChatGPT can support these collaborations by offering real-time suggestions or augmenting idea refinement without replacing human creativity. This aligns with findings that emphasize the interplay between peer collaboration and creative thinking in dynamic learning environments [77,78].

Finally, it’s true that based on this study’s results, AI-powered personalized learning, like ChatGPT can boosts engagement and creativity but can weaken critical thinking if overused. Educators must ensure students use AI for feedback, not as a replacement for independent thought [3,78]. Privacy and data security require strong safeguards, such as anonymizing inputs and limiting data storage. As AI becomes more prevalent in education, institutions must enforce clear policies to protect academic integrity and build trust [78]. By addressing these risks, AI can enhance learning without compromising ethics.

## 8. Conclusion, limitation and future research

This study provides significant insights into the transformative potential of integrating ChatGPT-driven personalized learning, Bloom’s Taxonomy, and peer collaboration to cultivate creative thinking skills in education. By systematically guiding learners through cognitive stages, ChatGPT facilitates higher-order thinking processes such as analysis, evaluation, and creation. The incorporation of peer learning interactions enhances collaborative creativity, enabling learners to co-construct knowledge and develop innovative solutions. These findings advance theoretical and practical understanding and highlights the dynamics between AI tools and human collaboration in education. However, the study also reveals the critical need for balance—while AI significantly enhances learning experiences and excessive reliance may compromise independent critical thinking.

This study has several limitations that highlight areas for improvement. The use of purposive sampling and self-reported data may reduce the accuracy and applicability of the findings. Differences in learners’ technological readiness affected how well they adapted to AI-driven learning, emphasizing the need to address digital literacy gaps. Challenges in peer collaboration also surfaced, as dominant voices sometimes dominated quieter participants and limits equal engagement. Additionally, the lack of long-term data makes it difficult to measure the lasting effects of AI-driven personalized learning and collaboration on creativity, underscoring the need for more in-depth research. Therefore, future research should address these limitations by using broader sampling methods and long-term studies to assess AI’s lasting impact on creativity and collaboration. Examining how structured facilitation improves peer interactions can help resolve group dynamics issues. Researchers should also explore the ethical and practical challenges of AI in education, including data privacy and equal access, to ensure fair and secure learning environments. By filling these gaps, future studies can enhance our understanding of AI’s role in education while promoting its responsible and inclusive use.

## Supporting information

S1 File Appendix Table A. (DOCX)
