# Supplementary material for: Redefining creativity in AI era: Bloom’s taxonomy framework and peer interaction elevate higher-order thinking skills
Source: PLoS One. 2026 Oct 14;21(10):e0356609. doi: 10.1371/journal.pone.0356609 (PMC13667817; doi:10.1371/journal.pone.0356609)
Supplement: Supplementary file 1 [file pone.0356609.s001.docx]

Appendix Table A

| Constructs | Items |
| --- | --- |
| Personalized Learning  Zhang et al. (2022) | ChatGPT aligns learning with my goals  ChatGPT provides diverse learning resources  ChatGPT tracks my strengths and weaknesses  ChatGPT supports my unique learning needs  ChatGPT encourages exploration of new topics |
| Bloom Taxonomy  Krathwohl (2002) | *Recognizing*  ChatGPT helps me to identify key concepts during learning  I use ChatGPT to retrieve specific information when needed  *Understand*  ChatGPT explains complex concepts in ways I can understand  ChatGPT provides examples that help me understand abstract ideas  ChatGPT helps me compare different concepts or approaches  ChatGPT provides me detailed explanations for questions I ask  *Apply*  I use ChatGPT to execute known methods efficiently  ChatGPT helps me apply knowledge to new and unfamiliar scenarios  *Analyze*  I use ChatGPT to identify key components in complex topics  ChatGPT helps me organize information into logical structures  *Evaluate*  ChatGPT helps me verify the accuracy of my solutions  ChatGPT helps me assess the quality of proposed solutions  *Create*  I use ChatGPT to brainstorming solutions for complex problems  ChatGPT helps me create effective plans for learning tasks |
| Creative Thinking Skills  Sternberg & Rainbow Project Collaborators (2006) | I demonstrate originality in my academic tasks by applying ChatGPT’s suggestions.  I can modify existing ideas or approaches using ChatGPT to fit new needs.  I use ChatGPT to inspire highly creative ideas for my work.  I demonstrate originality in group activities by leveraging ChatGPT’s capabilities.  I use ChatGPT to explore unique ways of completing my assignments or projects. |
| Peer Learning Interaction  Hwang et al. (2018) | Collaboration is successful when conducting learning tasks using ChatGPT.  Useful and sufficient information is provided during ChatGPT-enhanced learning activities.  Communication is effective when ChatGPT supports collaborative discussions.  Tasks are completed efficiently with tailored suggestions and feedback from ChatGPT.  Tasks are divided effectively based on abilities with ChatGPT's support. |
